# Effects of temperament during handling and social rank on the blood biochemical parameters of common eland (*Taurotragus oryx*)

**DOI:** 10.1007/s11259-024-10296-1

**Published:** 2024-01-23

**Authors:** A. S. Musa, J. Kundankumar, T. Needham, R. Kotrba, V. Ny, J. Consolacion, F. Ceacero

**Affiliations:** 1 Department of Animal Science and Food Processing, Faculty of Tropical AgriSciences, Czech University of Life Sciences Prague, Kamýcká 129, Praha-Suchdol, 165 00 Czech Republic; 2 Department of Ethology, Institute of Animal Science, Přátelství 815, Prague 10- Uhříněves, 104 00 Czech Republic

**Keywords:** Domestication, Physical restraining, Routine handling, Social rank, Welfare

## Abstract

Large herbivores are subject to handling and social stress in captivity. These may affect blood biochemical values, which motivated this research. Twelve healthy common eland (*Taurotragus oryx*) were monitored for 12 months. The animals were handled monthly, and blood samples were collected. Samples from every second month were analysed for 14 blood biochemical parameters. Temperament throughout the handling, as the summation of various behavioural responses, was calculated as a proxy of the stress generated during handling. Social behaviour was recorded each month, and the agonistic interactions were used to calculate the social rank, which was considered a proxy of social stress. Generalised Linear Mixed Models were designed to test the effects of temperament and social rank on the blood biochemical parameters while keeping sex, age, body condition, and body weight as covariates. The results show that the temperament during handling influences blood levels of albumin, alkaline phosphate, blood urea nitrogen, glucose, total bilirubin, and total protein; however, social rank has little influence, affecting just albumin. The ranges observed in the values of these biochemical parameters were still within their reference intervals, implying the absence of pathology or physiological problems during the study. The results suggest that blood biochemical values of physically restrained common eland should be carefully interpreted, even in animals already habituated to routine handling. On the contrary, social rank has low effects on the blood biochemical parameters.

## Introduction

The number of non-domesticated ungulate species kept in captivity for conservation or production purposes is steadily increasing. These species are more susceptible than domesticated ones to handling and social stress (Hemsworth 2003), which may lead to severe effects on their physiology and health. Several parameters are influenced by handling in semi-domesticated species, like blood sodium concentrations in reindeer (Hyvarinen et al. 1976), glucose (GLU) and phosphorus (P) in woodland caribou (Karns and Chrichton 1978), or several haematological parameters in red deer (Ceacero et al. 2018). On the contrary, animals often get habituated to routine handling (Ceacero et al. 2014; Musa et al. 2021); however, these stressors can go beyond their coping level, resulting in impaired reproductive function, immunity, growth, and increased frequencies of stereotyped behaviours (Koolhaas et al. 1999). Thus, this is the basis of the first hypothesis in this study: that the stress during handling influences the blood biochemical parameters of semi-domestic common eland.

Stress can also be induced by social dominance interactions resulting from physical confrontation during conspecific competition, particularly in captivity (Brakes 2019). In such conditions, animals compete to increase their resource-holding potential, which results in agonistic interactions potentially leading to injuries (Wirtu et al. 2004; Bica et al. 2020). The effects of these stressful agonistic interactions on physiological responses have been well-studied in domestic goats, where high- and low-ranked does produce less milk (Barroso et al. 2000). In red deer, social status influences the haematological parameters through immunosuppression (Hjarvard et al. 2009; Ceacero et al. 2018). On this background, we also hypothesised that the stress derived from the social rank of each animal in the herd would influence the blood biochemical parameters of common eland.

The common eland (*Taurotragus oryx*) has been described as an ideal antelope for domestication due to its large body mass, ease of habituation to handling, and meat production potential (Woodford 2000). Depending on their exposure to handling, they might still get stressed while handling for health inspection, management, and research purposes. Moreover, few published studies have focused on the blood biochemical of common eland in the wild and captivity thus far, with few reference values available (Pospisil et al. 1984; Vahala et al. 1989). In addition, these studies utilised different immobilisation methods, leading to discrepancies. Hence, this study also aims to establish reference intervals of the blood biochemical parameters for common eland in captivity, where it is possible to control for confounding factors such as age, sex, body weight, and body condition. Overall, the present study aims to examine the effects of the stress during handling (measured through the temperament during handling) and the social stress (measured through the social rank) on the blood biochemistry parameters of common eland and contribute toward establishing reference intervals for the species.

## Materials and methods

### Experimental site and animal husbandry

The research was conducted at the Czech University of Life Sciences Prague (CZU) Common Eland Research Facilities. Animals are managed in an intensive system in a 450 m^2^ barn with free access to 2.25 ha paddocks (low stocking density) and a complete feed mixture diet provided *ad libitum* inside the barn throughout the year (Musa et al. 2021). The animals in this study were managed in the same herd throughout the study. The animals are routinely handled every month; for this study, samples collected every second month for one year were selected.

The Czech Republic Ministry of Agriculture accredited all experimental procedures (clearance no. 63479_2016-MZE-17,214), and ethical clearance was obtained from the Czech University of Life Sciences Animal Welfare and Clearance Committee (no. CZU 20/19). Routine handling in a custom-designed squeeze chute system with a raceway involved body weight and condition measurement, blood collection, recording of the temperament (as described in Musa et al. 2021), and recording social interactions two days after the handling. Twelve healthy animals with 135.1 ± 2.25 kg initial body weight, 3.8 ± 0.1 body condition score (BCS, following the 1 to 5 scale for red deer; Audigé et al. 1998), and age 9.2 ± 1.5 months, consisting of six females six and males, were chosen for this study.

### Assessment of the stress during handling

The stress during handling was measured as the behavioural response during handling by a trained researcher (temperament score, a proxy for handling stress; Mahre et al. 2015; Musa et al. 2021; Parham et al. 2021). The final score is the sum recorded at each step of the handling, being 0 for the less stressed animals and 17 for the most stressed ones. Then, the score is divided by 17 to get computed to a 0–1 range for easier interpretation. Since the behavioural response during the immobilisation phase was especially important for this study, this variable (scored as calm/0, nervous/1, panicked/2 as described in Musa et al. 2021) was also separately considered in the statistical analyses.

### Social interactions and social rank

Social interactions were monitored by a trained researcher in order to obtain a social rank, which was later used as a proxy for social stress (Mahre et al. 2015; Musa et al. 2021; Parham et al. 2021). *Ad libitum* sampling (Altmann 1974) was used to continuously observe agonistic interactions for five hours (0800–1300) each month. Initiators and recipients of agonistic behaviours were recorded, including *Threatening* (with head or horns), *Passing* (moving away when another animal is passing), *Wrestling* (locking or clashing of horns), *Pushing* (with head or horns) and *Yielding* (butting another herdmate). Four commonly used dominance indices were computed using DomiCalc, a specialised software for the study of social interactions matrices (Schmid and de Vries 2013): David’s score, Clutton-Brock index, Proportion of dominations, and the Inconsistencies and strength of inconsistencies method (I&SI).

### Blood samples processing

Immediately after an animal is restrained in the squeeze chute, blood is collected from the *vena jugularis*. The area is disinfected, and blood samples were gently drawn using 18-gauge Vacuette® sterile needles with an adapter into a heparinised tube (Vacuette® Li-Heparin 8 ml). After collection, the tube was gently vortexed to avoid coagulation. The samples were then transferred to the laboratory in an ice-cold box and left to settle for at least 45 min before centrifugation at 2800 rpm for 15 min. The supernatant plasma sample was pipetted into Eppendorf tubes and stored below − 18 °C until analysis.

Plasma samples from every second month were analysed at the Laboratory of Animal Science (Faculty of Tropical AgriSciences, CZU), making 68 samples (four samples could not be collected). The frozen samples were thawed, vortexed, centrifuged at 12,000 RCF for 2 min, and analysed for blood biochemical parameters: albumin (ALB), globulin (GLOB), total proteins (TP), creatinine (CREA), blood urea nitrogen (BUN), alanine aminotransferase (ALT), alkaline phosphatase (ALP), lactate dehydrogenase (LDH), amylase (AMYL), glucose (GLU), calcium (Ca), phosphorus (P), cholesterol (CHOL), and total bilirubin (TBIL). Analyses were done using a VetTest® Chemistry Analyser (IDEXX Laboratories, Westbrook, Maine, USA) with a standard commercial kit commonly designed for a general determination of the health status of mammals (GPH – General Health Profile; IDEXX Laboratories, Westbrook, Maine, USA).

### Statistical analyses

Kolmogorov-Smirnov, visual examination of the histograms, and Q-Q plots were used for examining the normality of the variables analysed. Spearman´s ranked correlation showed a strong correlation for the two temperament variables (ρ = 0.412, *p* < 0.001) and very strong for the four social rank indices (all ρ > 0.802 and *p* < 0.001). Thus, Principal Component Analysis using Varimax rotation was used for both groups of variables to obtain a single factor for each one. For temperament, the selected factor explained 70.4% of the variance in the original variables. For social rank, the selected factor explained 74.8%. These factors were normally distributed and were used in further analyses. Generalised Linear Mixed Models tested the effect of temperament and social ranks on the 14 blood biochemical markers studied. A repeated measures structure was used, with individuals as subjects and months as a repeated measure. Sex, body weight, body condition, and age in days were also included in the models as covariates. All models were designed with normal distribution and identity link function, given the normality of all the variables used. Lack of collinearity was confirmed in the five continuous variables (temperament, social rank, body weight, BCS, and age) through the Variance Inflation Factor, which was always less than 5, as suggested (Kock and Lynn 2012). SPSS ver. 28.0 (IBM, Armonk, NY, USA) was used for all the analyses.

## Results

**Fig. 1:**
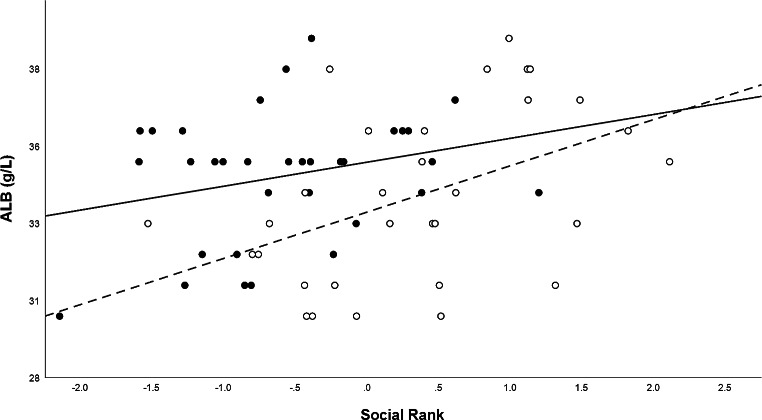
Changes in ALB concentrations (g/L) of captive common eland along the social rank gradient, separated by sex (black dots and full line for females; white dots and truncated line for males)

The blood biochemical results were within the expected range (ZIMS 2022) for all the animals (Table 1). Four samples were excluded as they had extreme values, which might have resulted from inadequate sample handling. As expected, most of the blood biochemical markers studied were, to a certain degree, affected by general characteristics like sex, age, body weight, and BCS (Table 2). When controlling for these factors, the first hypothesis was confirmed: temperament during handling, as a proxy of the stress during handling, affected several blood biochemical markers. ALP and BUN were higher in animals with a calm temperament, while ALB, GLU, TBIL, and TP were lower in these animals. On the contrary, the second hypothesis was not confirmed since social rank, as a proxy of social stress, only affected ALB. Males had a higher, but not significant, average social rank than females (t=-5.185, *p* = 0.197). Thus, the effect of social rank on increasing ALB values can be considered independent of sex, which was also a significant variable explaining ALB values (Fig. 1).

**Table 1:** Descriptive statistics of the blood biochemical parameters for common eland obtained in this study, compared with reference intervals (ZIMS 2022)

Biochemical parameters	N	Mean	Median	Maximum – Minimum	Reference Interval (95%)	Reference Interval (ZIMS 2022)
ALB (g/L)	64	34.2	35.0	30.0–39.0	30.0–39.0	13.0–42.9
ALP (U/L)	64	96.0	87.0	43.0–206.0	43.6–202.3	36–367
ALT (U/L)	64	23.5	23.0	10.0–38.0	10.6–36.1	1–25
AMYL (U/L)	63	455	451	301–600	326–597	30–750
BUN (mmol/L)	64	5.95	5.90	4.20–8.00	4.33–7.75	2.9–12.1
Ca (mmol/L)	63	2.36	2.41	1.59–2.80	1.62–2.67	1.7–2.8
CHOL (mmol/L)	64	2.12	2.05	1.52–3.08	1.62–3.02	0.65–2.58
CREA (µmol/L)	64	127	125	91–172	92–163	35–265
GLOB (g/L)	64	41.4	41.0	35.0–48.0	35.6– 47.38	3–59
GLU (mmol/L)	64	4.45	4.45	2.64–6.66	2.75–6.24	3.61– 15.65
P (mmol/L)	64	2.52	2.27	1.25–5.96	1.31–5.48	0.94–3.88
TBIL (µmol/L)	64	4.08	4.00	1.00–8 − 00	1.00–8.00	1.7–23
TP (g/L)	64	75.6	76.0	67.0–85.0	67.0–83.75	47–93
LDH (u/L)	63	399.4	367.3	213.7–872.0	216.4–825.9	352–1794

**Table 2:** General Linear Mixed Models showing the factors affecting the blood biochemical parameters of common eland. The table shows the β coefficients and the *p*-values (in brackets) obtained for the full models. *P*-values under 0.1 are shown, while the rest are just indicated as not significant

Blood parameters	Sex^1^	Age	Body condition	Body weight	Social rank	Temperament
ALB (g/L)	2.140 (< 0.001)	-6.056 (< 0.001)	0.909 (ns)	0.038 (0.031)	0.817 (0.024)	0.892 (< 0.001)
ALP (U/L)	44.495 (ns)	-1.391 (ns)	-22.767 (0.020)	0.764 (0.003)	-0.510 (ns)	-8.401 (0.053)
ALT (U/L)	0.446 (ns)	0.019 (ns)	0.235 (ns)	0.044 (ns)	0.011 (ns)	1.474 (ns)
AMYL (U/L)	-2.455 (0.090)	131.227 (< 0.001)	-44.846 (0.006)	-1.351 (< 0.001)	-2.707 (ns)	-0.940 (ns)
BUN (mmol/L)	0.110 (ns)	-0.438 (ns)	0.675 (0.013)	-0.006 (ns)	0.042 (ns)	-0.413 (< 0.001)
Ca (mmol/L)	0.193 (0.005)	-0.384 (0.004)	-0.249 (0.002)	0.008 (< 0.001)	-0.007 (ns)	0.001 (ns)
CHOL (mmol/L)	0.077 (ns)	0.221 (ns)	-0.055 (ns)	-0.001 (ns)	-0.018 (ns)	-0.004 (ns)
CREA (µmol/L)	7.247 (0.065)	20.764 (0.010)	20.480 (< 0.001)	-0.147 (ns)	-0.055 (ns)	-2.163 (ns)
GLOB (g/L)	-0.862 (ns)	4.104 (0.017)	1.122 (ns)	-0.081 (< 0.001)	0.058 (ns)	0.093 (ns)
GLU (mmol/L)	-0.019 (ns)	-1.048 (0.001)	0.185 (ns)	-0.007 (ns)	0.027 (ns)	0.308 (< 0.001)
P (mmol/L)	-0.650 (0.012)	0.660 (ns)	0.600 (0.043)	-0.023 (0.001)	0.171 (ns)	0.025 (ns)
TBIL (µmol/L)	-0.533 (ns)	-0.921 (ns)	0.337 (ns)	-0.010 (ns)	0.231 (ns)	0.619 (0.006)
TP (g/L)	0.294 (ns)	-2.432 (ns)	2.564 (0.059)	-0.035 (ns)	-0.190 (ns)	1.385 (0.006)
LDH (u/L)	-215.985 (ns)	-491.339 (ns)	349.656 (ns)	5.598 (ns)	-112.700 (ns)	179.961 (ns)

^1^ Male as a category of reference

Temperament and social rank did not influence other blood biochemical parameters, including AMYL, Ca, CREA, GLOB, and P. Nevertheless, certain variability was explained by the controlling factors sex, age, body condition, and body weight. On the other hand, ALT, CHOL, and LDH were not affected by any of the studied factors.

## Discussion

Blood biochemical parameters, like electrolytes, enzymes, fat, protein, and energy-related metabolites, provide information about an animal’s metabolic, nutritional, and health conditions, thereby providing insight into the welfare status of an animal. This study shows that all the values were within the reference intervals as compared to available data (ZIMS 2022), but these markers became altered in response to the studied factors when controlling for others like age, sex, weight, and body condition, which are already known to affect blood biochemistry (Kumar et al. 2022). Temperament, as a proxy of the stress during handling, influenced some enzymes, proteins, and energy metabolites, but not electrolytes, while social rank, as a proxy of social stress, only affected ALB. Other factors such as age, sex, body weight, and body condition were found to play a significant role in the blood biochemical profile of animals and their response to stress. These variables were included in the present study to increase the robustness of the models and confirm the effects independently of these confounding factors. Since these factors have already been reported in many studies, they are not further discussed.

Under acute stress, animals mobilise glucose to power the body to overcome the impact of such stressors. Handling commonly induces stress, resulting in behavioural displays that can be measured. This temperament during handling influenced many of the studied blood metabolites of common eland, with the effect on glucose being one of the most interesting. Glucose concentrations were higher in those animals that were more nervous during handling, confirming our hypothesis about handling stress and previous findings (Kumar et al. 2022). Acute phase proteins such as ALB also increased with stronger temperament reactions, as did TP, while no effect was observed for GLOB. These show directional changes in the body’s immunity under stress and align with the findings of Marco and Lavin (1999), which show higher values in serum ALB and TP concentrations when animals were physically immobilised compared to chemical immobilisation. On the other hand, Murata et al. (2004) have reported not an increase but a decrease in ALB under different challenges, including stress; therefore, this effect needs further attention.

Elevated BUN concentrations are due to the high production of ammonia in the rumen, largely affected by the protein content in the diet. Calm animals showed higher BUN concentrations. However, the reason for the increase cannot be directly linked to the temperament during handling but suggests that calmer animals may have better access to feed resources. TBIL is another marker that increased with handling stress. It is a marker for cholestasis, which accompanies liver problems. A potential reason for the observed pattern can be the instantaneous breakdown of red blood cells, which are released during the acute response to stress stimuli to promote the efficient transportation of oxygen required for cellular catabolism (Marco and Lavin 1999). The cellular metabolism can increase exponentially in stressed animals, requiring enzymes to facilitate the success of this mechanism, although the temperament during handling did not influence the enzymes analysed in this study. A possible explanation for the normal enzyme concentrations is the absence of clinical pathologies and the not-so-acute stress that might appear after physical injury. Other studies have recorded higher ALT, AST, CK, and LDH concentrations in stressed animals during physical immobilisation, transportation, or handling for examination (Ali et al. 2006). Similarly, significant changes in CREA concentrations can be attributed to muscle damage (Kumar et al. 2022), which was not evident in the present study. At the same time, minerals such as Ca and P are maintained by hormonal regulation, which is usually in equilibrium in healthy individuals.

Social stress is a common phenomenon in social species. Occasionally, the agonistic interactions for access to resources may be even deadly due to the dominant-submissive relationships (Creel et al. 1996). Indeed, the effects of social stress on blood haematology have already been reported (Ceacero et al. 2018). This was the basis of our second hypothesis, which was not confirmed since social rank only influenced ALB among all of the 14 blood biochemical parameters analysed. ALB concentrations typically increase under dehydration; however, water is offered *ad libitum*, and no fluid loss, such as diarrhoea, was observed during the study. The lack of differences in other parameters can be attributed to the general harmony in the social group, as no serious agonistic interaction was recorded during the study. In a similar work, Tuchscherer et al. (1998) also found no differences in GLU, TP, triglyceride and ALP concentrations between dominant and subordinate pigs. However, significant differences appeared in TP, GLU, ALP and cortisol concentrations after adding new individuals to the group. Thus, the results suggest that the impact of social stress would be less important in well-established social groups with stable linearity and hierarchy. Another explanation may be linked to the fission-fusion social system of the species, which may make social rank not so important as in other social ungulates.

In summary, this study confirms that temperament during handling affects the blood biochemical profile of captive common eland, even if the animals involved are well used to routine handling and several individual variables were controlled for. On the contrary, effects caused by social rank were mainly rejected, except for ALB. Considering the handling technique and how habituated the animals are to it should help veterinarians interpret their results during clinical diagnosis. This study also verifies the reference values under a large sample size, establishing the reference values of 14 different blood biochemical parameters for the species. Future studies involving larger herds and species with different social systems are recommended.

The results obtained confirm the suitability of the species for domestication. Their social system allows keeping different groups with different structures, sizes and compositions and modifying them without increasing the social stress of the animals and raising welfare problems. The species can also be easily habituated to routine handling practices (Musa et al. 2021) necessary in intensive captive breeding programs, with handling having just certain stress effects on the animals, as expected, but not so strong as to challenge their welfare.

## Data Availability

The data that support the findings of this study are available from the corresponding author upon reasonable request.
